# Induced Pluripotent Stem Cell-Derived Brain Endothelial Cells as a Cellular Model to Study *Neisseria meningitidis* Infection

**DOI:** 10.3389/fmicb.2019.01181

**Published:** 2019-05-29

**Authors:** Sara F. Martins Gomes, Alexander J. Westermann, Till Sauerwein, Tobias Hertlein, Konrad U. Förstner, Knut Ohlsen, Marco Metzger, Eric V. Shusta, Brandon J. Kim, Antje Appelt-Menzel, Alexandra Schubert-Unkmeir

**Affiliations:** ^1^Institute of Hygiene and Microbiology, University of Würzburg, Würzburg, Germany; ^2^Institute of Molecular Infection Biology (IMIB), University of Würzburg, Würzburg, Germany; ^3^Helmholtz Institute for RNA-based Infection Research (HIRI), Helmholtz Centre for Infection Research (HZI), Würzburg, Germany; ^4^ZB MED, Information Centre for Life Sciences, Cologne, Germany; ^5^TH Köln, University of Applied Sciences, Faculty of Information Science and Communication Studies, Cologne, Germany; ^6^Chair Tissue Engineering and Regenerative Medicine, University Hospital Würzburg, Würzburg, Germany; ^7^Fraunhofer Institute for Silicate Research ISC, Translational Center Regenerative Therapies (TLC-RT), Würzburg, Germany; ^8^Department of Chemical and Biological Engineering, University of Wisconsin–Madison, Madison, WI, United States

**Keywords:** *Neisseria meningitidis*, meningococcus, bacteria, stem cells, blood-cerebrospinal fluid barrier, blood-brain barrier, brain endothelial cells

## Abstract

Meningococcal meningitis is a severe central nervous system infection that occurs when *Neisseria meningitidis* (*Nm*) penetrates brain endothelial cells (BECs) of the meningeal blood-cerebrospinal fluid barrier. As a human-specific pathogen, *in vivo* models are greatly limited and pose a significant challenge. *In vitro* cell models have been developed, however, most lack critical BEC phenotypes limiting their usefulness. Human BECs generated from induced pluripotent stem cells (iPSCs) retain BEC properties and offer the prospect of modeling the human-specific *Nm* interaction with BECs. Here, we exploit iPSC-BECs as a novel cellular model to study *Nm* host-pathogen interactions, and provide an overview of host responses to *Nm* infection. Using iPSC-BECs, we first confirmed that multiple *Nm* strains and mutants follow similar phenotypes to previously described models. The recruitment of the recently published pilus adhesin receptor CD147 underneath meningococcal microcolonies could be verified in iPSC-BECs. *Nm* was also observed to significantly increase the expression of pro-inflammatory and neutrophil-specific chemokines *IL6*, *CXCL1*, *CXCL2*, *CXCL8*, and *CCL20*, and the secretion of IFN-γ and RANTES. For the first time, we directly observe that *Nm* disrupts the three tight junction proteins ZO-1, Occludin, and Claudin-5, which become frayed and/or discontinuous in BECs upon *Nm* challenge. In accordance with tight junction loss, a sharp loss in *trans-*endothelial electrical resistance, and an increase in sodium fluorescein permeability and in bacterial transmigration, was observed. Finally, we established RNA-Seq of sorted, infected iPSC-BECs, providing expression data of *Nm*-responsive host genes. Altogether, this model provides novel insights into *Nm* pathogenesis, including an impact of *Nm* on barrier properties and tight junction complexes, and suggests that the paracellular route may contribute to *Nm* traversal of BECs.

## Introduction

*Neisseria meningitidis* or meningococcus (*Nm*) is a Gram-negative human-exclusive pathogen that asymptomatically colonizes the upper respiratory tract of 10–40% of the world population (Rouphael and Stephens, 2012). In susceptible individuals, *Nm* infection is a leading cause of purpura fulminans and meningitis. Meningitis is a disease that is still associated with high mortality despite administration of modern antibiotic therapy (Rouphael and Stephens, 2012). Invasive meningococcal disease initiates when *Nm* crosses the epithelium of the nasopharynx and reaches the bloodstream, where it is able to resist complement-mediated killing and proliferate (Quagliarello and Scheld, 1992). Binding of *Nm* to the endothelium of brain microvessels that compose the meningeal blood-cerebrospinal fluid barrier (b-CSF) and blood-brain barrier (BBB) is a crucial step in disease progression (Mairey et al., 2006; Weller et al., 2018). Binding of type IV pili to host receptor CD147 triggers the formation of highly ordered CD147/β2-adrenergic receptor clusters assembled by the scaffolding protein α-actinin-4 (Maïssa et al., 2017). The activation of this receptor promotes the recruitment of the polarity complex to the bacterial adhesion site together with adherens and tight junction proteins, leading to the opening of a paracellular route that may facilitate bacterial passage (Coureuil et al., 2009; Bernard et al., 2014). Previous work has demonstrated that *Nm* is internalized by endothelial cells in membrane-bound compartments referred to as *Neisseria*-containing vacuoles, presumed to offer a protective niche for intracellular multiplication and potentially serve as a vehicle for transcytosis (Nikulin et al., 2006). Therefore, the primary transit route of BECs by *Nm* has yet to be determined, highlighting the need for further investigation.

Though initial host-pathogen contact is thought to be type IV pili-dependent, the interaction and invasion of the b-CSF barrier is greatly supported by other virulence factors such as colony opacity associated proteins Opa and OpcA (Virji et al., 1993), and recently identified minor adhesion and adhesion-like proteins [reviewed in Hung and Christodoulides (2013)]. Previous studies using an immortalized line of human brain microvascular endothelial cells (HBMECs) also reported that *Nm* infection induces the release of pro-inflammatory cytokines involved in neutrophil and monocyte recruitment (Sokolova et al., 2004; Dick et al., 2017).

The b-CSF barrier is one of five main interfaces identified in the adult brain and BECs are presumed to be the first obstacle that meningococci must breach in order to reach the leptomeninges (Weller et al., 2018). BECs are highly specialized cells that maintain cerebral homeostasis and protect the brain from toxic compounds and pathogens (van Sorge and Doran, 2012; Weller et al., 2018). BECs accomplish this through their selectively permeable and barrier-forming phenotype, that arises from the presence of complex tight junctions, efflux transporters and highly regulated receptor-mediated transcytosis (Engelhardt and Sorokin, 2009). Studies with immortalized HBMECs and other endothelial cell lines have elucidated many of the known *Nm*-BEC interactions (Schubert-Unkmeir, 2017). However, immortalized cell lines do not retain important phenotypes of BECs such as strong barrier properties, namely high *trans-*endothelial electrical resistance (TEER), responsiveness to other cell types of the neurovascular unit, and expression of important tight junction markers (Weksler et al., 2005; Eigenmann et al., 2013).

Advances in human stem cell technologies have provided the prospect of utilizing highly scalable human systems to model specific cell types such as BECs (Lippmann et al., 2012). BECs generated from induced pluripotent stem cells (iPSC) exhibit advantages over current *in vitro* models of BECs as they retain expected brain phenotypes and functional features while also being of human origin. In addition, iPSC-BECs possess significant barrier properties, reaching TEER values over 2500 Ω × cm^2^ (Lippmann et al., 2014). Finally, iPSC-BECs have been shown to respond to other cell types of the neurovascular unit that help regulate barrier function (Lippmann et al., 2012, 2014; Appelt-Menzel et al., 2017; Canfield et al., 2017).

The human specificity of *Nm* and the lack of robust BEC phenotypes in presently utilized models emphasize the need for new models possessing properties more consistent with BECs *in vivo*. Here, we demonstrate the usefulness of iPSC-BECs *in vitro* to study *Nm* infection. Additionally, we profiled host expression patterns in response to *Nm* infection by specifically selecting invaded iPSC-BECs and sequencing their transcriptome. Altogether, this model provides novel insights into *Nm* pathogenesis, including an impact of *Nm* on barrier properties and tight junction complexes, and suggests that the paracellular route may contribute to *Nm* traversal of BECs.

## Results

### *Nm* Interacts With iPSC-Derived BECs

**FIGURE 1:**
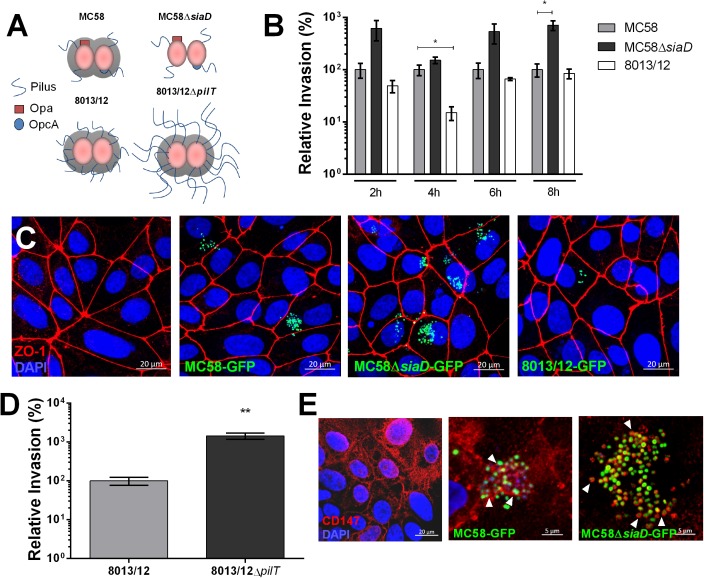
Characterization of *Nm* interaction with iPSC-BECs. **(A)** Schematic cartoon of *Nm* strains used. MC58 is a serogroup B strain, MC58Δ*siaD* is an isogenic non-capsulated mutant, 8013/12 is a serogroup C strain and 8013/12Δ*pilT* is a highly piliated mutant. **(B)** Gentamicin protection assay of *Nm* on iPSC-BECs showing invasion of MC58Δ*siaD* and 8013/12 relative to MC58 into iPSC-BECs at the indicated time points and Multiplicity of Infection (MOI) of 10. **(C)** Confocal microscopy images of iPSC-BECs infected with the *Nm* strains mentioned in **(B)**, at 4 h p.i. and MOI 100. Image is a maximum image projection. Scale bar = 20 μm. **(D)** Gentamicin protection assay showing invasion of Δ*pilT* mutant relative to WT 8013/12 *Nm* strains into iPSC-BECs at 4 h p.i. and MOI 10. For **(B,D)** data is presented as mean ± S.E.M of three independent experiments done in technical duplicate and triplicate, respectively. Student’s *t*-test was used to determine significance. ^∗^*p* < 0.05; ^∗∗^*p* < 0.01. **(E)** Immunofluorescence staining showing areas of recruitment of receptor CD147 (red) around MC58 and MC58Δ*siaD* colonies (green) highlighted with white arrow heads in iPSC-BECs at 4 h p.i. and MOI of 100. Scale bar = 5 μm.

To investigate whether *Nm* interacts with iPSC-BECs, iPSCs were differentiated to BECs according to previously published methods (Lippmann et al., 2012, 2014; Stebbins et al., 2016; Appelt-Menzel et al., 2017). We observed expected barrier properties as measured by high TEER and marker expression, such as CD31 and Glut-1, as previously described (Stebbins et al., 2016; Supplementary Figure 1). Monolayers were infected with two wild-type (WT) and two mutant *Nm* strains all expressing a variety of characterized virulence factors (Figure 1A). Gentamicin protection assays were used to estimate the relative invasion of *Nm* into iPSC-BECs. Capsule-deficient MC58Δ*siaD* was observed to be more invasive than its WT strain, and levels were consistent to previous reports with immortalized BECs, where no differences between growth curves were observed (Unkmeir et al., 2002; Johswich et al., 2013; Figure 1B). Similar patterns were observed by confocal microscopy, where MC58Δ*siaD* was relatively more abundant compared with the parent WT strain (Figure 1C). Previous work with immortalized cells have demonstrated the importance of the pilus in mediating bacterial invasion into host BECs (Hoffmann et al., 2001). Although no significant difference in invasion could be observed in a pilus-deficient *ΔpilE* mutant (Supplementary Figure 2B), we found that the pilus-overexpressing *ΔpilT* mutant is hyper invasive in iPSC-BECs compared to invasion rates of the WT strain, suggesting a role for the pilus during infection (Figure 1D). Absolute values of invasion of these strains are shown in Supplementary Figure 2, as well as the respective growth curves. Binding of type IV pilus to BECs is hypothesized to be a critical aspect of *Nm* infection preceding the translocation of the b-CSF barrier (Coureuil et al., 2012). CD147 was recently described as the type IV pilus receptor in a study conducted with immortalized endothelial cells (Bernard et al., 2014). Immunofluorescence staining of CD147 during infection with GFP-expressing MC58 and MC58*ΔsiaD* strains shows close proximity of this receptor at sites of bacterial attachment similar to the previous report (Bernard et al., 2014; Figure 1E). Taken together, these results demonstrate that various strains and mutants of *Nm* can interact with iPSC-BECs following similar patterns and phenotypes to previously described models, and that iPSC-BECs possess an important receptor for virulence.

### *Nm* Infection Leads to Barrier Permeability

**FIGURE 2:**
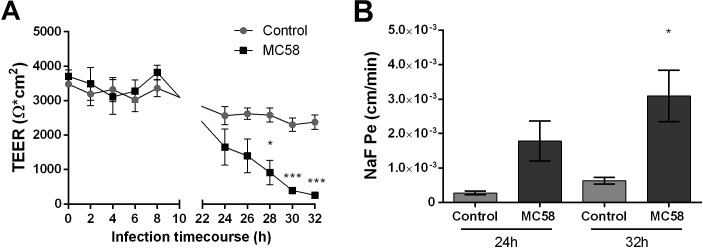
*Neisseria meningitidis* infection impacts barrier properties of iPSC-BECs. **(A,B)** iPSC-BEC monolayers seeded onto 0.4 μm pore size transwells with or without *Nm* challenge at MOI 10 were used for **(A)** monitoring of TEER values (Ω × cm^2^) over a time course of 32 h and **(B)** determination of NaF permeability coefficient (cm/min) at 24 and 32 h p.i. The data are represented as mean ± S.E.M. of three independent experiments each done in technical triplicate. Student’s *t*-test was used to determine significance. ^∗^*p* < 0.05; ^∗∗∗^*p* < 0.001.

To assess if *Nm* challenge impacts barrier integrity of iPSC-BECs, the TEER profile of mock and infected monolayers was measured over time. At 24 h a trend for decreasing TEER could be observed in infected transwells, which became significant at 28 h (Figure 2A). At 32 h TEERs of infected monolayers dropped 90% when compared to controls, suggesting that *Nm* causes barrier disruption at late infection time points. To further establish that *Nm* infection impairs barrier function, paracellular permeability to sodium fluorescein (NaF) was quantified (Stebbins et al., 2016). In support of the TEER dynamics, an increase in NaF permeability was observed at 24 h, which became significant at 32 h (4.9-fold higher for infected cells when compared to controls) (Figure 2B). These data suggest that *Nm* infection impairs barrier function of iPSC-BEC monolayers by increasing barrier permeability.

### *Nm* Infection Results in Tight Junction Disruption

**FIGURE 3:**
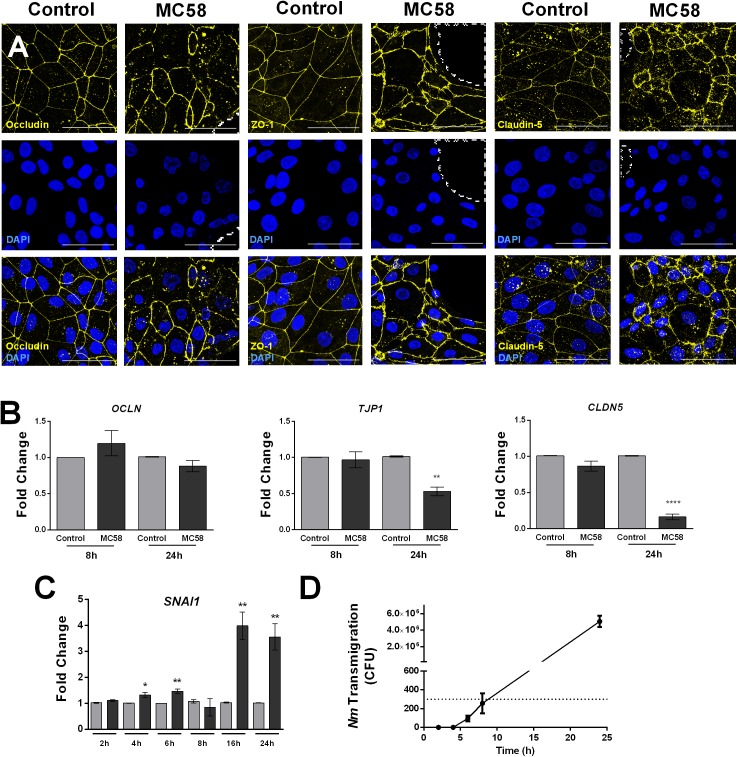
*Neisseria meningitidis* induces tight junction disruption and bacterial transmigration in iPSC-BECs. **(A)** Confocal microscopy images showing tight junction staining for Occludin, ZO-1 and Claudin-5 in iPSC-BECs seeded on ibidi microscopy slides with and without *Nm* challenge at MOI 10 at 24 h of infection. Areas outlined with dashed lines indicate gaps between cells. Image is a maximum image projection. Yellow = tight junctions, Blue = DAPI. Scale bar = 50 μm. **(B,C)** qPCR showing relative expression of **(B)** tight junction-coding genes *OCLN*, *TJP1*, and *CLDN5* at 8 and 24 h p.i. and **(C)** tight junction repressor gene *SNAI1* over a time course of 24 h, performed on iPSC-BECs with (dark gray bars) or without (light gray bars) *Nm* challenge at MOI 10. **(D)** Transmigration of *Nm* across monolayers of polarized iPSC-BECs seeded onto 3 μm pore size transwells at MOI 10. The number of CFUs traversing the layer was determined by assessing bacteria in the basolateral chamber at 2, 4, 6, 8, and 24 h. For B, C and D the data are presented as mean ± S.E.M. of three independent experiments done in triplicate. Student’s *t*-test was used to determine significance. ^∗^*p* < 0.05; ^∗∗^*p* < 0.01; ^∗∗∗∗^*p* < 0.0001.

To observe if there are changes in host tight junctions during infection, immunostaining for tight junction components ZO-1, Occludin, and Claudin-5 was conducted. No changes in the levels and localization of tight junction proteins could be detected at 8 h and 16 h p.i. (Supplementary Figures 3A,B). However, at 24 h p.i. gaps between cells and frayed junctions were observed. Junction discontinuity could be observed for Occludin and Claudin-5 but not ZO-1, however, ZO-1 appeared to be frayed (Figure 3A). No changes in protein content occurred at early time points, however, a trend for a decrease of ZO-1, Occludin, and Claudin-5, could be observed at 32 h of infection (Supplementary Figure 3C). Interestingly, an additional band at lower molecular weight was observed for Occludin, which may correspond to a cleavage product generated upon *Nm* infection, as previously described in immortalized HBMECs (Supplementary Figure 3C; Schubert-Unkmeir et al., 2010). A higher molecular weight band for Occludin could be observed at 24 and 32 h p.i., possibly corresponding to post-translational modifications (Ni et al., 2017). Consistent with the protein data, regulation of tight junction expression at the mRNA level in infected monolayers was not observed at 8 h p.i., whereas at 24 h *TJP1* and *CLDN5* expression significantly decreased (Figure 3B). Previous work suggests that bacteria can disrupt tight junctions in BECs through the upregulation of host Snail-1 (*SNAI1*), a transcriptional repressor of tight junction components (Kim et al., 2015, 2017; Yang et al., 2016). To assess if this transcription factor is upregulated during *Nm* infection, qPCR for *SNAI1* was conducted. We observed that upregulation of *SNAI1* occurs as early as 4 h p.i. in infected cultures, although it is notably higher at later time points (Figure 3C). Taken together, these findings suggest that tight junctions are disrupted in BECs during *Nm* infection.

To determine if the observed junctional disruption contributes to *Nm* crossing through iPSC-BECs, transmigration studies were carried out. Bacteria could not be detected in the basolateral chamber of infected transwells before 6 h of infection, and remained below the detection limit of the assay at 6 and 8 h. At 24 h, the number of CFUs crossing the monolayer drastically increased when compared to 8 h of infection (Figure 3D). Together, these results demonstrate that *Nm* causes tight junction disruption of iPSC-BECs possibly through upregulation of host *SNAI1*. *Nm*-induced tight junction re-arrangements coincide with a dramatic increase in *Nm* traversal through the endothelial barrier.

### *Nm* Infection Activates iPSC-BECs

**FIGURE 4:**
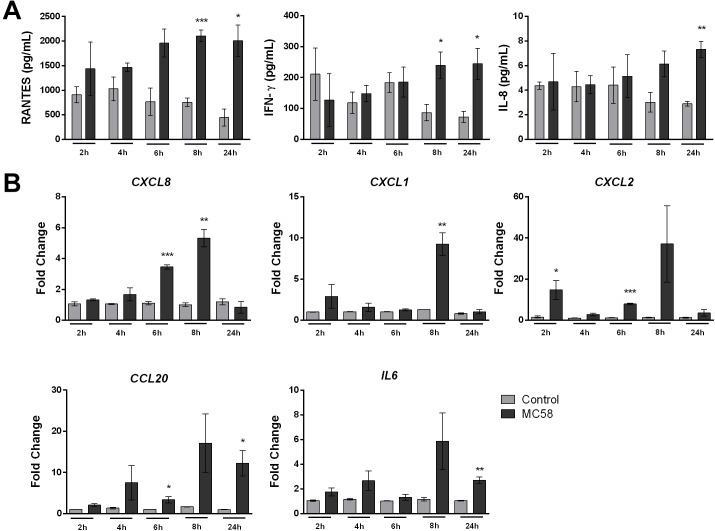
*Neisseria meningitidis* infection leads to activation of iPSC-BECs. **(A)** Detection of RANTES, IFN-γ and IL-8 on supernatants of mock- (light gray bars) and bacteria-infected (dark gray bars) iPSC-BECs by Luminex bead-based multiplex assays during a time course of 2, 4, 6, 8 and 24 h of infection. **(B)** Quantitative PCR showing relative expression of *CXCL8, CXCL1, CXCL2*, *CCL20*, and *IL6* transcripts during a time course of 2, 4, 6, 8, and 24 h of infection for mock- (light gray bars) and *Nm*-infected (dark gray bars) monolayers. Data are presented as mean ± S.E.M of three independent experiments done in duplicate **(A)** or triplicate **(B)**. Student’s *t*-test was used to determine significance. ^∗^*p* < 0.05; ^∗∗^*p* < 0.01; ^∗∗∗^*p* < 0.001.

Meningitis disease progression is characterized by the inflammation of the meninges, presumed to be provoked by BECs (Tunkel and Scheld, 1993). To investigate whether iPSC-BECs are activated upon *Nm* infection, a panel of 14 cytokines/chemokines was selected for Luminex bead-based multiplex assays. Our results show that RANTES and IFN-γ are significantly more abundant in the supernatants after 8 and 24 h of *Nm* infection (Figure 4A). Although a statistically significant increase in IL-8 levels in supernatants derived from infected monolayers was observed at 24 h p.i., it should be noted that the magnitude of secretion (8 pg/mL) is very low compared with other cell types (Dick et al., 2017; Figure 4A). Similar trends were observed for supernatants collected from monolayers infected with *Nm* strains MC58Δ*siaD* and 8013/12 (Supplementary Figure 4A). qPCR on RNA from infected cultures confirmed upregulation of *CCL5* (RANTES) and *IFNG* (INF-γ) at 24 h p.i. (Supplementary Figure 4B). No detection or detection below the standard limit was observed for IL-6, IL-1β, Gro-α, GM-CSF, TNF-α, ICAM-1, and vWF-A2 (data not shown). No difference between control versus MC58 was observed for VCAM-1, E-Selectin, and Gro-β (Supplementary Figure 4C). In contrast, time-dependent increase of IL-6 and IL-8 could be detected for immortalized HBMECs infected with MC58 and MC58Δ*siaD* (Supplementary Figure 4D), as described previously (Dick et al., 2017). Activation of iPSC-BECs in response to *Nm* infection was further assessed with qPCR of neutrophilic chemoattractants and activators *CXCL8*, *CXCL1*, *CXCL2*, *CCL20*, and *IL6* gene expression. Our results show that these important neutrophilic factors are upregulated during the time course of infection (Figure 4B). Overall, our data suggest that iPSC-BECs can sense and respond to bacteria and are activated upon *Nm* challenge through the upregulation of some pro-inflammatory factors.

### RNA-Seq of *Nm*-Infected iPSC-BECs

**FIGURE 5:**
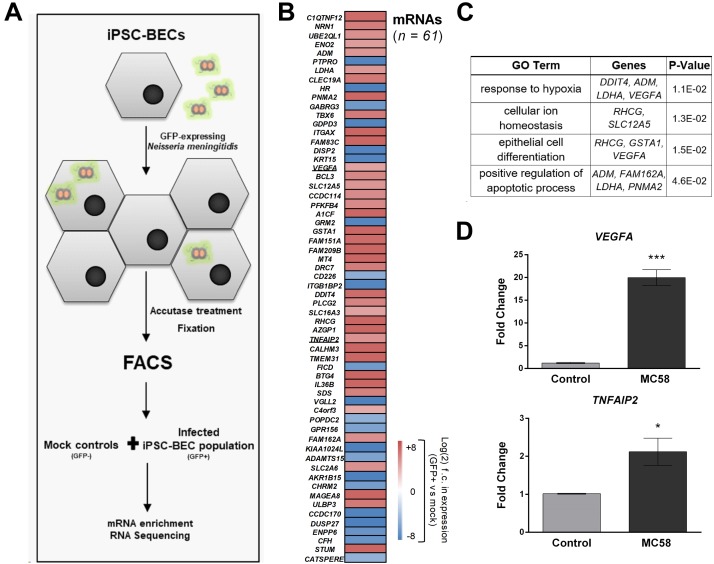
RNA-Seq of sort-enriched, infected iPSC-BECs. **(A)** Experimental workflow. iPSC-BECs were infected with *Nm*-GFP at MOI 10, detached through Accutase treatment, and fixed in RNA*later*©. An outline of the gating strategy is provided in Supplementary Figure 5A. Mock controls without *Nm* challenge were sorted and collected in parallel (i.e., GFP-negative gated population P3 of mock, Supplementary Figure 5A). Total RNA was extracted from each sample and polyadenylated transcripts were converted into cDNA libraries and sequenced to ∼25–30 million reads/library. **(B)** Heat map showing differentially expressed mRNAs between GFP-positive, infected cells and mock controls (without *Nm* challenge) at 24 h of infection. Plotted are all genes that were significantly differentially expressed (adjusted *P*-value < 0.1; DESeq2). Sequencing data was derived from two biological replicates. **(C)** Gene Ontology analysis of differentially expressed mRNAs shown in **(B)**, acquired with DAVID bioinformatics resources. Enriched GO terms with *P*-value < 0.05 are shown as well as the differentially expressed mRNAs corresponding to the respective pathway. **(D)** Validation of RNA-Seq data by qPCR. *VEGFA* and *TNFAIP2* mRNA levels were increased at 24 h after MC58 infection (MOI 10) as compared to mock-treated cultures. RNA was isolated from monolayers of iPSC-BECs without sorting. The data are presented as mean ± S.E.M of three independent experiments done in triplicate. Student’s *t*-test was used to determine significance. ^∗^*p* < 0.05; ^∗∗∗^*p* < 0.001.

Above, we characterized the response of bulk cultures of *Nm*-challenged iPSC-BECs, i.e., without discriminating between invaded and non-invaded, bystander cells. To specifically track transcriptomic changes originated by the population of invaded host cells, we enriched infected (GFP-positive) from non-infected (GFP-negative) iPSC-BECs at 24 h of *Nm* infection using fluorescence-activated cell sorting (FACS) as previously described (Westermann et al., 2016; Figure 5A and Supplementary Figure 5A). From the collected *Nm*-invaded cell population, we isolated total RNA and converted the polyadenylated transcripts (i.e., human mRNAs and polyadenylated long non-coding RNAs) into cDNA libraries. RNA-Seq analysis led to the identification of a total of 100 differentially expressed genes [61 mRNAs and 39 non-coding RNAs (ncRNAs), Figure 5B and Supplementary Figure 5B] as compared to mock-treated control cells. Of the 61 mRNAs, 22 were downregulated while 39 were upregulated relative to the uninfected control. Interestingly, while several of the upregulated mRNAs (e.g., *TNFAIP2*, *BCL3*) were previously reported as *Nm*-induced host transcripts in a human blood-cerebrospinal fluid barrier model by microarray analysis (Borkowski et al., 2014), others had not been identified in this previous screen. Gene Ontology (GO) analysis of the induced mRNAs using DAVID (Huang et al., 2009) yielded significantly enriched (*P*-value < 0.05) GO terms, generally related to cellular stress and hypoxia (Figure 5C). We sought to validate some of these results through independent qPCR on RNA samples from bulk (unsorted) cultures. Specifically, the mRNA encoding vascular endothelial growth factor A (VEGFA), which was previously implicated as a target of meningitic *Escherichia coli* to disrupt the BBB (Yang et al., 2016), was upregulated by invaded iPSC-BECs, as judged from RNA-Seq (23-fold vs. mock). This strong induction could be confirmed by qPCR (Figure 5D). Likewise, induced expression of *TNFAIP2* mRNA, encoding the proinflammatory TNFα-induced protein 2 known to be involved in the response to *Nm* infection (Linhartova et al., 2006; Borkowski et al., 2014), was observed by both RNA-Seq and qPCR (Figure 5B,D). Together, this global expression dataset confirms previous observations, but may also serve as a valuable resource for future studies.

## Discussion

Knowledge of the interaction between *Nm* and BECs is critical for understanding meningococcal meningitis progression and for the development of novel therapeutic intervention. Until now, modeling this interaction has proved difficult due to the intrinsic properties of BECs and the human-exclusive tropism of *Nm*. *In vitro* studies of *Nm* interaction with brain capillaries have largely relied on human bone marrow-derived endothelial cells (Hoffmann et al., 2001; Bernard et al., 2014) and immortalized cell lines such as the brain microvascular endothelial cell line hCMEC/D3 (Weksler et al., 2005; Coureuil et al., 2009, 2010; Bernard et al., 2014) and HBMECs (Unkmeir et al., 2002; Nikulin et al., 2006). While HBMECs lack Claudin-5 expression, hCMEC/D3 cells express Claudin-5 but lack continuous Occludin and exhibit relatively low TEER (Weksler et al., 2005; Eigenmann et al., 2013). Alternatively, humanized mice have been used *in vivo*, but these must rely on known interactions and/or have limited translatability (Johswich et al., 2013; Melican et al., 2014). Here, we demonstrate a significant technical advance in the modeling of *Nm* interaction with human BECs by using iPSC-BECs. This model overcomes drawbacks of current models, as (i) it is of human origin while still being highly scalable, and (ii) it retains important BEC properties, such as the expression of tight junction complexes that translate into high barrier potential. Moreover, this model has been used to model the BBB in the past. However, here we propose to model the BECs of the meningeal b-CSF barrier. BECs that constitute the capillaries located within the subarachnoid space possess high barrier properties and maintain BEC phenotypes, without the support of neurovascular unit cell types such as pericytes and astrocytes (Cassella et al., 1997; Møllgård et al., 2017; Rua and McGavern, 2018; Weller et al., 2018). Our data suggest that the iPSC-BEC model can be used to study *Nm* attachment, invasion, disruption of tight junctions and endothelial barrier, immune activation as well as transcriptome changes upon infection. Moreover, our results correlate well with previous work regarding invasion of several *Nm* strains and mutants to immortalized cell lines, and our model expresses the identified pilus receptor, CD147, which localizes with meningococcal colonies (Figure 1E).

As a cellular model for studying central nervous system pathogens, iPSC-BECs have recently been utilized for the study of Zika virus and Group B *Streptococcus* infection (Kim et al., 2017; Alimonti et al., 2018). Here we used a retinoic acid enhanced iPSC-BEC model that achieves physiological TEERs that allowed us to observe that prolonged *Nm* infection leads to significant decreases in BEC TEER, and increased paracellular permeability to the small molecule sodium fluorescein and to *Nm* (Figure 2, 3D). At the tight junction level, these modifications of barrier functionality were associated with the formation of highly frayed junctions, junctional discontinuity, and opening of gaps between cells. The generation of gaps between *Nm*-infected cells and subsequent opening of the paracellular route has been suggested to contribute to *Nm* crossing of BECs (Coureuil et al., 2009). Importantly, our data correlate bacterial transmigration with tight junction disruption (Figure 3D,A), supporting the hypothesis that *Nm* may utilize the paracellular route for BEC crossing. Although transcellular crossing at early time points of infection may occur, the observed CFU values were below the detection limit of the technique.

A particular strength of our model is the expression and localization of tight junction components, in particular Claudin-5 (Helms et al., 2015; Greene et al., 2019). Here we show for the first time disruption of Claudin-5 upon *Nm* challenge (Figure 3A). Our results also support previous observations that *Nm* induces cleavage of Occludin leading to the generation of a 50-kDa fragment detected by Western blotting (Schubert-Unkmeir et al., 2010; Supplementary Figure 3C). In agreement with the mentioned observations of tight junction disruption, *Nm* infection of iPSC-BECs upregulated host *SNAI1* (Snail-1), a transcriptional repressor of tight junction components operating in the infection mechanisms of Group B *Streptococcus* (Kim et al., 2015, 2017). Yang et al., 2016 also correlated Snail-1 expression with the upregulation of VEGFA upon *in vivo* and *in vitro E. coli* K1 challenge, causing increased BBB permeability. In the present study, our RNA-Seq data showed upregulation of *VEGFA* in response to *Nm* infection, which was confirmed by qPCR (Figure 5B,D). Thus, the role of VEGFA in host responses to *Nm* may include bacterial hijacking mechanisms for BEC disruption, which may be worth further investigation.

Bacterial meningitis progression is characterized by the recruitment of highly activated leukocytes into the CSF, which is presumed to be triggered by the inflammatory activation of BECs (Tunkel and Scheld, 1993). Using iPSC-BECs we were able to detect RANTES and IFN-γ secretion upon meningococcal infection, both previously detected in the CSF of patients suffering from bacterial meningitidis (Glimåker et al., 1994; Pashenkov et al., 2002). In addition, our data indicate that iPSC-BECs respond to *Nm* infection by upregulating *IL6* (IL-6)*, CXCL8* (IL-8), *CXCL1* (Gro-α), *CXCL2* (Gro-β), and *CCL20* (MIP3A) at the mRNA level (Figure 4B). Interestingly, the observed transcriptional upregulation did not accompany secretion of IL-6, and of IL-8 at physiological levels. Our data support published findings regarding the modeling of Group B *Streptococcus* infection using a similar model of iPSC-BECs that reported transcriptional evidence for immune activation (Kim et al., 2017). Taken together, we observe activation of iPSC-BECs through the transcriptional upregulation of chemokines and cytokines in addition to the detection of IFN-γ and RANTES in the supernatant, however, further investigation may be needed to understand the regulation of cytokine secretion in this model.

Finally, RNA-Seq of sorted-enriched, infected iPSC-BECs was established to track transcriptomic changes in the host upon *Nm* internalization. For this, we built upon a fixation and FACS protocol previously established for a *Salmonella*-based infection system (Westermann et al., 2016). To our knowledge, this is the first time that RNA-Seq has been implemented to the study of *Nm*-BEC interactions. GO term analysis of the resulting data suggested that *Nm* infection elicits hypoxia, cellular ion homeostasis and apoptotic responses in iPSC-BECs. Activation of hypoxia-inducible factor 1 (HIF1) is a well-known cell response to hypoxic conditions induced by sepsis and LPS stimulation (Peyssonnaux et al., 2007; Werth et al., 2010; Textoris et al., 2012) and HIF1 signaling was proposed to mediate VEGFA induction during *Clostridium difficile* infection (Huang et al., 2019). Although the impact of hypoxia and oxidative stress during *Nm* infection in particular has been poorly described, evidence of *Nm*-induced oxidative DNA damage in infected cells has been previously observed (Oosthuysen et al., 2016). By contrast, positive regulation of apoptosis is a well-known transcriptomic host response to *Nm* infection (Constantin et al., 2002; Schubert-Unkmeir et al., 2007).

Notably, analysis of individual genes upregulated in the gene set revealed a few genes that have been associated to *Nm* infection but poorly explored thus far. In particular, *Integrin Subunit Alpha X* (*ITGAX*) encoding for CD18/CD11c, is upregulated by 76-fold (Figure 5B). CD18/CD11c has been described to be expressed in human umbilical vein endothelial cells (Langeggen et al., 2002) and to function as a phagocytic receptor for *Nm* when transfected into CHO cells (Jones et al., 2008). *TNFAIP2*, upregulated 33-fold (Figure 5B), is phosphorylated upon LPS stimulation (Chevrier et al., 2011) and its expression is linked to increased mortality in patients experiencing septic shock (Thair et al., 2016). Finally, *CFH* coding for complement factor H, a key immune regulator of the host immune response that is hijacked by *Nm* through binding to factor H-binding protein (fBHP) (Welsch and Ram, 2008), is downregulated in host iPSC-BECs (Figure 5B). Overall, our transcriptomic analysis of specifically infected iPSC-BECs seems to be useful at late time points of infection for the detection of potential key host genes regulated in response to *Nm*. Still, host transcriptomic responses to *Nm* infection are likely to be time-dependent and may vary considerably from early to late infection stages. The low infection rate at early time points (Supplementary Figure 5A) and current sensitivity of bulk RNA-Seq technologies impeded similar transcriptomics at the 4 and 8 h time point. However, ongoing progress in (single-cell) RNA-Seq techniques (Saliba et al., 2014) should allow for transcriptome profiling during earlier stages of *Nm* infection in future efforts. Likewise, future studies should be directed toward a more comprehensive transcriptome profiling of our infection model, wherein not only host expression changes during infection, but also that of intracellular *Neisseria* may be measured [(a so-called “dual RNA-Seq” approach see Westermann et al. (2012)].

Our study demonstrates that iPSC-BECs are a potentially superior model to study BEC-*Nm* interaction. We observed that *Nm* infection disrupts barrier properties and results in the destruction of the major BEC tight junction components, previously unable to be measured due to lack of expression/localization in previous models. This destruction greatly increases bacterial transmigration through iPSC-BEC monolayers.

## Materials and Methods

### Bacterial Strains and Cell Lines Used

*Neisseria meningitidis* strain MC58 was used unless otherwise specified. MC58 serogroup (Sg) B strain, sequence type (ST)-74 [ST-32 clonal complex (cc)], kindly provided by E. R. Moxon (McGuinness et al., 1991). *Nm* serogroup C strain 8013/clone12 [SgC ST-18cc (ST-177)] (Nassif et al., 1993). Non-capsulated mutant of strain MC58 (MC58Δ*siaD*) as described (Unkmeir et al., 2002). Meningococci were cultured on Columbia Agar with 5% sheep blood (COS medium, bioMérieux, #43049) and incubated at 37°C with 5% CO_2_ overnight. Subsequent liquid culturing was performed in proteose-peptone medium (PPM) plus 1% Kellogg’s supplement I and II (i.e., PPM+ medium). Human iPSC cells IMR90-4 (WiCell) were maintained on Matrigel^®^ (BD Biosciences, #354230)-coated 6-well plates (Sarstedt) in mTeSR^TM^1 medium (STEMCELL Technologies, #05850) or StemFlex medium (Gibco, # A3349401) with daily medium changes. Passaging was performed with Versene (Gibco, #15040-033) or Dispase (Gibco, #17105-041) at a splitting ratio of 1:6 or 1:12. All as described in Stebbins et al. (2016).

### Generation of iPSC-BECs From iPSCs

IMR-90-4 iPSC cells were differentiated according to previously published methods (Lippmann et al., 2012, 2014; Stebbins et al., 2016; Appelt-Menzel et al., 2017, 2018). Briefly, single iPSC cells were disassociated with Accutase (Sigma-Aldrich, #A6964) and cultured at a density of 0.75–1 × 10^4^ iPSC/cm^2^ on Matrigel-coated 6-well plates or T75 flasks (Sarstedt). After 3–4 days, media was changed to Unconditioned Medium [DMEM/F-12 (Gibco, #31330-038) + 20% KnockOut^TM^ Serum Replacement (Gibco, #10828028) + 1% MEM Non-Essential Amino Acids (Gibco, #11140-035) + 0.5% Glutamax (Gibco, #3505-038) + 0.1 mM β-Mercaptoethanol (Sigma-Aldrich, #M3148)] with daily medium changes for 6 days. The medium was then changed to EC medium [human Endothelial serum-free medium (hESFM, Gibco, #11111-044) supplemented with 1% platelet-poor plasma derived bovine serum (PDS, Alfa Aesar, #J64483AE), 20 ng/mL hbFGF (PeproTech, #100-18B) and 10 μM all-*trans* Retinoic Acid (Sigma-Aldrich, #R2625)] for 2 days. BECs were then purified through seeding into collagen IV (Sigma-Aldrich, #C5533)- and fibronectin (Sigma-Aldrich, #F2006)-coated 48-well plates (Thermo-Nunc, #150687), 12-well format transwells (Corning, #3460 or #3462) or chambered coverslip slides. The day after BEC purification, medium was changed to EC medium without RA and hbFGF. Infection experiments were done at day 10 of differentiation.

### Infection Assays

The medium of confluent iPSC-BECs at day 10 of differentiation was changed to hESFM medium supplemented with 10% Human Serum (HS, TCS biosciences, #CR100-500), followed by infection with *Nm* strains with MOI of 10 (unless specifically noted) for the time indicated, no media change was conducted as described in Kim and Schubert-Unkmeir (2019). Bacteria grown overnight in solid culture were resuspended in 10 mL PPM+medium and incubated in a shaker at 37°C, 200 rpm for 60 to 90 min. Bacteria concentration was estimated by OD_600_ measurements. Bacteria or PPM+ medium alone (i.e., mock-infected controls) were then inoculated into differentiated iPSC-BEC monolayers. Infection was carried out at 37°C and 5% CO_2_ to a maximum of 32 h.

### Immunofluorescence and Microscopy

iPSC-BEC monolayers seeded onto 8-well ibidi μ-Slides (Ibidi, #80821) were washed with PBS and fixed with methanol or 3.7% PFA for 15 min. After washing, PFA-fixed cells were permeabilized with 0.1% Triton X-100 in PBS for 15 min. Washing was followed by blocking in 10% Fetal Calf Serum (Life Technologies, #10270) or 10% Goat Serum (Sigma-Aldrich, #G9023) in PBS for 1 h at RT. Primary antibodies [ZO-1 (1:100, Proteintech, #21773-1-AP), Claudin-5 (1:100, Abcam, #ab15106), Occludin (1:200, Thermo, #33-1500), and CD147 (1:100, Biorad, #MCA2882Z)] were incubated overnight at 4°C. After washing, incubation with secondary antibodies [Alexa 555-conjugated donkey anti-rabbit (Thermo, #A31572), Alexa 647-conjugated donkey anti-mouse (Thermo, #A31571) or Alexa 488-conjugated goat anti-mouse (Thermo, #A11001)] was done at room temperature for 1 h. Confocal microscopy images were acquired with a Zeiss LSM 780 microscope. ZEN software (version 2.3 blue edition) was used for image analysis.

### TEER Measurements, Paracellular Permeability Studies, and Bacterial Transmigration Assays

At day 10 of differentiation the electrical resistance of iPSC-BECs was measured using a Millicell ERS-2 instrument (Merck) as previously described (Stebbins et al., 2016). Infected or control transwells were used to measure TEER every 2 h and to estimate sodium fluorescein (NaF) permeability at 24 and 32 h p.i., as described (Stebbins et al., 2016). Transwells with pore size of 3 μm were used for bacterial transmigration studies. Briefly, at each mentioned time point, transwells were washed with PBS and moved to fresh EC medium for 30 min, after which 100 μL of basolateral medium were plated into 5% sheep blood agar plates for counting of colonies [method adapted from Coureuil et al. (2009)]. Limit of detection was determined by multiplying the minimum CFU possible to be measured on agar plates (15 CFU per 1.5 mL of basolateral medium) by the minimum CFU number recommended for usage in spread plate protocols for CFU enumeration by the American Society for Microbiology (Wise, 2006).

### Protein Collection and Western Blotting

iPSC-BEC monolayers were washed with ice-cold PBS three times, then total protein lysates were collected with lysis buffer [10 mM Tris–HCL pH 6.8, 100 mM NaCl, 1 mM EDTA, 10% Glycerol, 1% Triton^TM^ X-100, 0.1% SDS, 0.5% sodium deoxycholate, 2 mM Na_3_VO_4_, 50 mM Sodium Fluoride, 50 μg/ml Pefabloc (Carl Roth, #A154) and protease inhibitors (Roche, # 4693116001)]. After resuspension and incubation for 30 min on ice, lysates were centrifuged for 20 min at 12,000 *g* at 4°C. Total protein concentration was estimated using the Pierce BCA Protein Kit Assay (Thermo, # 23225) and 10 to 25 μg of protein were electrophoretically separated using 6 or 12% acrylamide gels. Following protein transfer to nitrocellulose membranes (GE Healthcare Life Sciences, # 10600001), blocking was performed with 5% skim milk with 0.1% Tween 20 in PBS solution for 1 h. Primary antibody incubation [Occludin (1:500), ZO-1 (1:1000), Claudin-5 (1:250), β-Actin (1:1000, Cell Signaling, #4967)] occurred overnight at 4°C. The following day, membranes were washed and incubated for at least 1 h with secondary antibody 1:10,000 Peroxidase AffiniPure Goat Anti-Mouse (Jackson ImmunoResearch, #115-035-044) or 1:5,000 Anti-Rabbit (Jackson ImmunoResearch, #115-035-006) at room temperature. Detected proteins were visualized using the Clarity Western ECL kit (Biorad, #170-5060).

### RNA Isolation and Quantitative PCR

iPSC-BEC monolayers were lysed with RNA lysis buffer and total RNA was recovered using the NucleoSpin kit (Macherey-Nagel, #740955). 500 ng of total RNA was used to synthetize cDNA with SuperScript^TM^ IV VILO^TM^ (Invitrogen, #11756050) and quantitative PCR data was collected with a StepOnePlus real-time PCR thermocycler (Thermo). Quantitative PCR for *CXCL1, CXCL2, CXCL8, CCL20, IL6, SNAI1* mRNAs and *18S* rRNA was performed using PowerUp SYBR Green (Thermo, #A25741) and primers formerly described (van Sorge et al., 2008; Rho et al., 2010), while TaqMan (Thermo, #4369016) was used for detection of *TJP1* (ZO-1; Hs0155186_m1), *CLDN5* (Claudin-5; Hs00533949_s1), *OCLN* (Occludin; Hs00170162_m1), *CCL5* (RANTES; Hs00982282_m1), *IFNG* (IFN-γ; Hs00989291_m1), *VEGFA* (Hs00900055_m1), *TNFAIP2* (Hs00969305_m1), and *18S* (Hs99999901_s1). Data are presented as fold change over *18S* using the cycle threshold (ΔΔCT) calculation.

### Cytokine/Chemokine Multiplex Bead Assays

Supernatants of iPSC-BEC monolayers of mock controls or cells infected with MC58, MC58Δ*siaD* or 8013/12 at 2, 4, 6, 8, and 24 h p.i were collected and tested for IL-1β, IL-6, IL-8, GM-CSF, Gro-α, Gro-β, TNF-α, IFN-γ, VCAM-1, ICAM-1, E-selectin, vWF-A2, MCP-1 and RANTES presence using a customized anti-human cytokine Luminex Multiplex Assay (R&D Systems), according to the manufacturer′s instructions. Baseline levels of all targets present in infection medium alone (hESFM supplemented with 10% HS) were subtracted from sample measurements for data analysis.

### FACS, RNA Extraction and DNase Treatment

Infection was carried out with GFP-expressing MC58 and mock-infected controls for 24 h p.i. for two independent experiments in iPSC-BEC monolayers previously seeded onto 6-well plates in hESFM + 10% HS. Cells were dissociated using Accutase for at least 20 min at 37°C and detached cells were centrifuged at 1200 rpm for 5 min. Cells were resuspended in RNA*later* solution (Qiagen, #76104) supplemented with 200 μg/mL gentamicin and stored at 4°C until the sorting day. Cells were pelleted (250 g, 5 min at RT) and resuspended in 300 to 1500 μL of ice-cold PBS for sorting. Cells were analyzed and sorted into GFP-negative and GFP-positive sub-populations using a FACSAria III device (BD Biosciences) at 4°C and using a 100 micron nozzle, as previously described (Westermann et al., 2016). Fractioned cells were lysed by resuspension in TRIzol (Invitrogen, #15596026) and RNA was precipitated through the TRIzol method. Briefly, resuspended cells and chloroform (0.2 mL/mL) were added to 5PRIME Phase Lock Gel Heavy tubes (Quantabio, #733-2478). The tubes were spun at 12,000 × g for 15 min at 4°C. Isopropanol (1 volume) and GlycoBlue (Ambion, #AM9516) were added to the obtained aqueous phase and the mixture was incubated at −20°C overnight. The mixture was then spun for 30 min at full speed and the pellet was rinsed with cold 70–75% ethanol. After air-drying for 10 min, the pellet was dissolved in RNase-free water by 5 min of incubation at 65°C and 850 rpm on a thermoshaker (Eppendorf). DNase I digestion (Fermentas) was carried out according to the manufacturer′s instructions. Total RNA was precipitated by phenol-chloroform extraction followed by ethanol precipitation. Briefly, 2.5 volumes of 30:1 RNA precipitation mix (EtOH: 3M NaOAC, pH 6.5) and Glycoblue were added to the aqueous fraction containing DNA-depleted RNA and precipitation was performed as described above.

### mRNA Enrichment, cDNA Library Generation and RNA-Seq

From the total RNA preparations, first-strand cDNA was synthesized using an oligo(dT)_25_ primer. After fragmentation, the Illumina TruSeq sequencing adapters were ligated in a strand-specific manner to the 5′ and 3′ ends of the cDNA fragments. The cDNA was amplified by PCR using a proofreading enzyme. The primers used for PCR amplification were designed for TruSeq sequencing according to the instructions of Illumina (the combined length of the flanking sequences was 136 bases). The final cDNA samples were analyzed on a Shimadzu MultiNA microchip electrophoresis system. For Illumina sequencing, cDNA libraries samples were pooled in equimolar amounts. The cDNA pools were size fractionated in the size range of 200–600 bp using a differential clean-up with the Agencourt AMPure kit. An aliquot of the size-fractionated cDNA pool was analyzed by capillary electrophoresis. Sequencing was performed on a NextSeq500 platform (Illumina) in single-end mode, for 75 cycles.

### Computational Analysis of RNA-Seq Data

Illumina reads were initially quality trimmed with a Phred quality score cut-off of 20 and afterward the adapter sequences were removed. Both trimming steps were done by cutadapt version 1.17 (Martin, 2011). For the poly(A) trimming, size filtering, and mapping, the RNA-Seq analysis tool READemption version 0.4.3 (Förstner et al., 2014) was used, which integrates the short read mapper segemehl (Hoffmann et al., 2009). Before mapping, poly(A) sequences were clipped from the reads. Afterward, all reads that had a length shorter than 20 nucleotides were discarded. The remaining reads were mapped, using segemehl’s split align feature, to the genomes of *Nm* MC58 (NCBI RefSeq accession number: NC_003112.2; as an additional quality control) and human (GENCODE release 28: GRCh38.p12). Reads with an accuracy equal or greater than 95% were kept for further analysis. Segemehl’s realigner lack (Otto et al., 2014) was used for remapping reads that previously could not be mapped due to multiple splice events. For the human-mapped reads, gene quantification was carried out by READemption (Förstner et al., 2014), while differential gene expression analysis was performed using DESeq2 version 1.20.0 (Love et al., 2014). Coverage files in wiggle (WIG) format were produced by READemption in a strand-specific manner, and normalized by the total number of aligned reads per organism multiplied by 1 million.

### Statistics

GraphPad Prism (Version 6.01) was used for statistical significance, for which a *P*-value of less than 0.05 was accepted. Student’s *t*-test was used for two-group comparisons. ANOVA was used for three-group comparisons.

## Data Availability

The RNA-Seq data discussed in this publication have been deposited in NCBI’s Gene Expression Omnibus (PMID: 11752295) and are accessible through GEO Series accession number GSE126449. Publicly available datasets were analyzed in this study. This data can be found here: https://www.ncbi.nlm.nih.gov/geo/query/acc.cgi?acc=GSE126449.

## Author Contributions

SMG, BK, AA-M, and AS-U contributed to the conception and design of the study. SMG performed the experiments and wrote the manuscript. SMG and BK designed the experiments and analyzed the data. TS, KF, and AW analyzed the RNA-Seq data. AS-U acquired funding. ES, BK, AA-M, MM, KO, TH, and AS-U provided reagents and/or resources. BK, MM, AA-M, and AS-U supervised the work. All authors contributed to the manuscript revision, and read and approved the submitted version.

## Conflict of Interest Statement

The authors declare that the research was conducted in the absence of any commercial or financial relationships that could be construed as a potential conflict of interest.
