# Supplementary figures and images for: Induced Pluripotent Stem Cell-Derived Brain Endothelial Cells as a Cellular Model to Study Neisseria meningitidis Infection
Source: Front Microbiol. 2019 May 29;10:1181. doi: 10.3389/fmicb.2019.01181 (PMC6548865; doi:10.3389/fmicb.2019.01181)

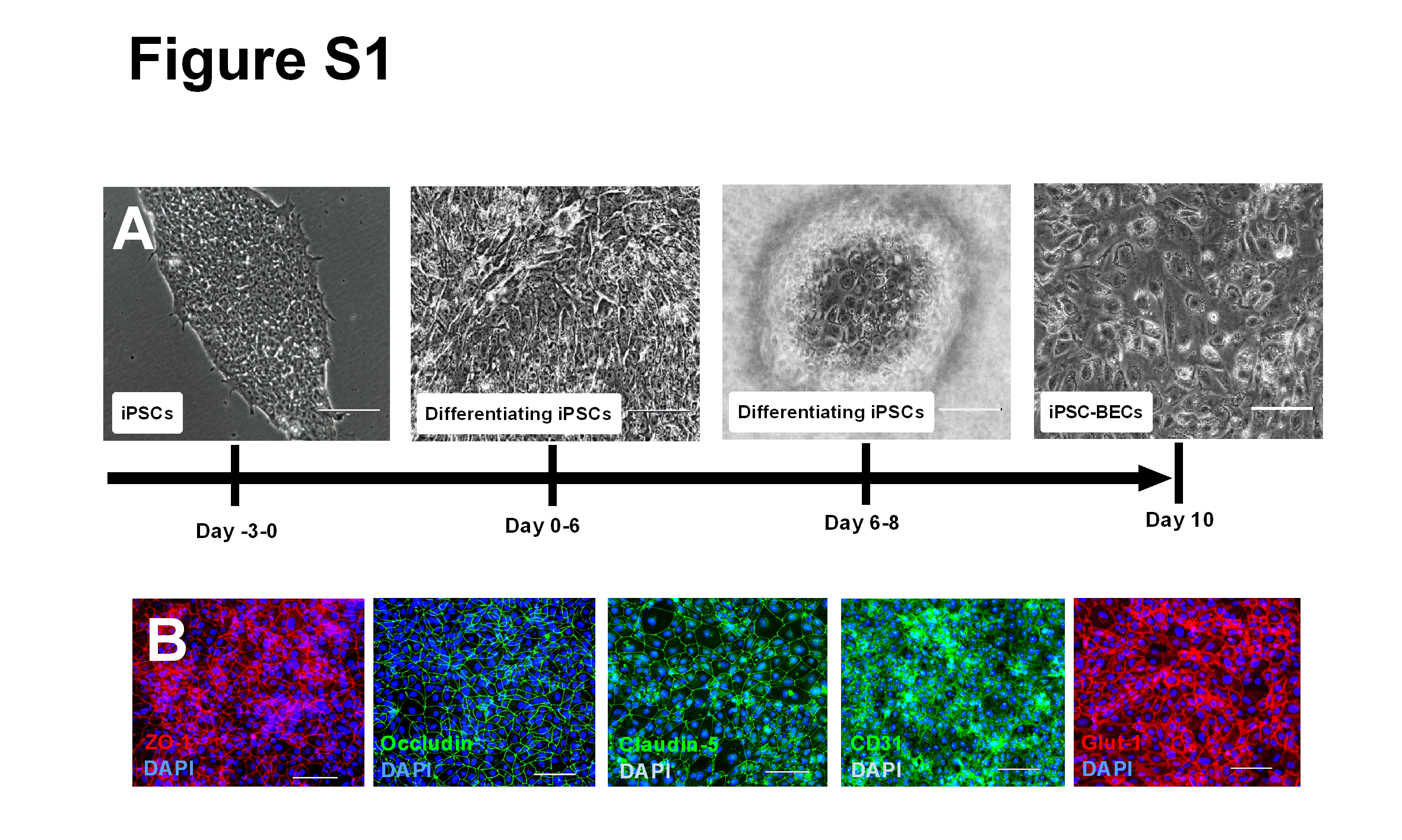

Supplement: Supplementary file 1 [file Image_1.TIF]

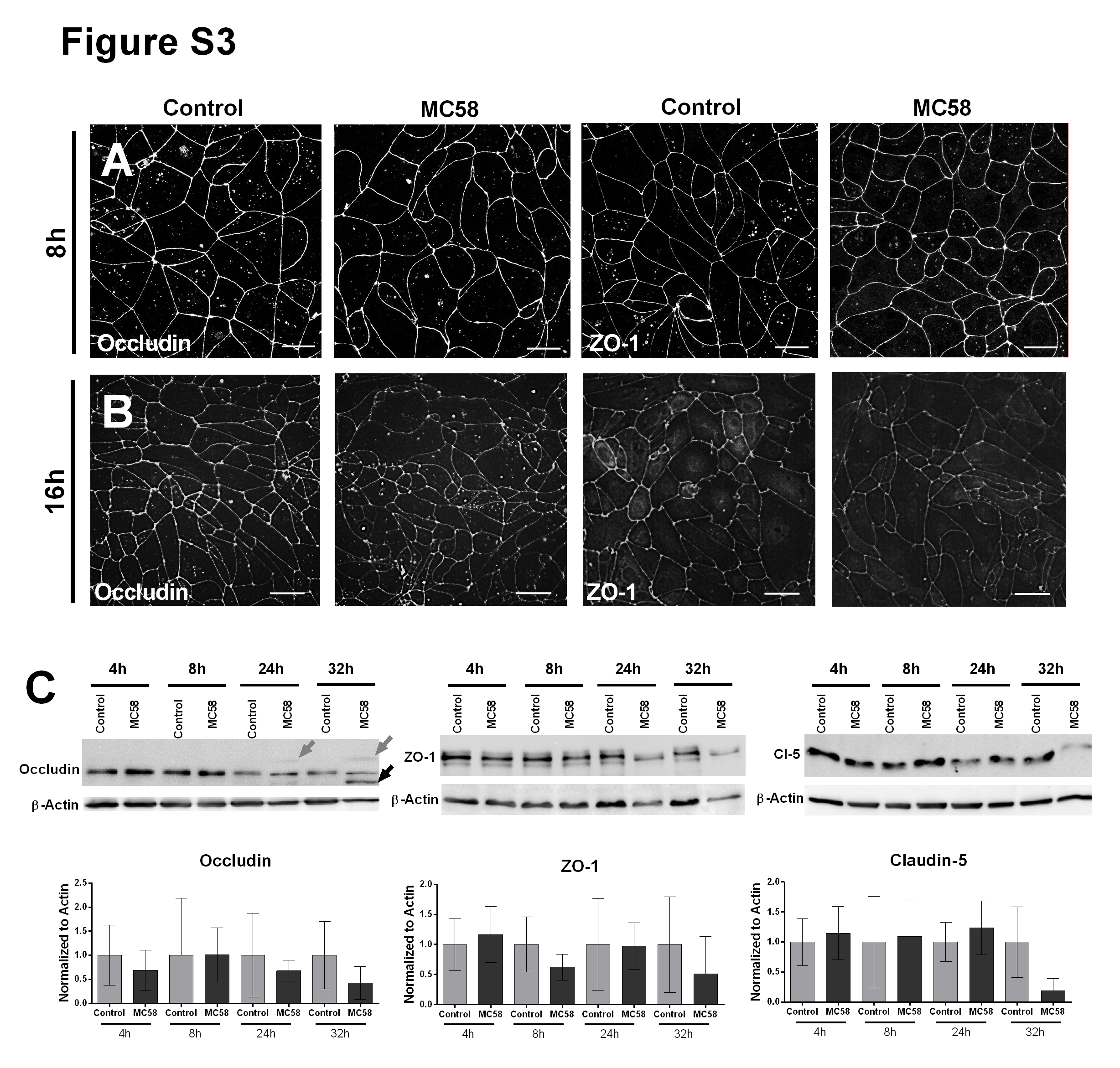

Supplement: Supplementary file 3 [file Image_3.TIF]
