# Supplementary material for: Induced Pluripotent Stem Cell-Derived Brain Endothelial Cells as a Cellular Model to Study Neisseria meningitidis Infection
Source: Front Microbiol. 2019 May 29;10:1181. doi: 10.3389/fmicb.2019.01181 (PMC6548865; doi:10.3389/fmicb.2019.01181)
Supplement: Supplementary file 6 [file Table_1.DOCX]

# Supplementary Figures

**Supplementary Figure 1. Generation of BECs from iPSCs.** A) iPSCs seeded onto matrigel-coated plates are induced to differentiate into BECs once the appropriate cell density is reached. BECs grow in pocket-like structures and are isolated from neighboring cells by purifying onto Collagen IV and Fibronectin at day 8 of the protocol. Cell morphologies during the differentiation process are similar to that observed in (Lippmann et al. 2012). The cells reach the highest TEER values at day 10 (see Fig 2A), when they are used for infection experiments. Scale bars = 100µm. (B) iPSC-BEC specific marker expression. At day 10 of differentiation, differentiated BECs express tight junction markers such as ZO-1, Occludin and Claudin-5, as well as CD31 and Glut-1. Red = ZO-1 or Glut-1. Green = Occludin, Claudin-5 or CD31. Blue = DAPI. Scale bars = 100 µm.

**Supplementary Figure 2. Characterization of *Nm* interaction with iPSC-BECs.** (A) Gentamicin protection assay of *Nm* on iPSC-BECs showing absolute invasion values of MC58, MC58Δ*siaD* and 8013/12 into iPSC-BECs at the indicated time points and MOI of 10. (B) Gentamicin protection assay showing absolute invasion of WT 8013/12 and Δ*pilE* and Δ*pilT* into iPSC-BECs at 4h p.i. and MOI 10. For A and B the data is presented as mean ± S.E.M of three independent experiments done in technical triplicate. (C) Growth curves of WT strain 8013/12 and the corresponding Δ*pilE* and Δ*pilT* mutants, obtained in PPM+ medium. No obvious differences were found between mutants and the corresponding parental strain. Student’s *t* test was used to determine significance in A. Two-way ANOVA was used to determine significance in B. *, p < 0.05; **, p < 0.01.

**Supplementary Figure 3. *Nm* infection on tight junction dynamics of iPSC-BECs.** (A and B) Images showing intact tight junction complexes after MC58 challenge using (A) Confocal Microscopy at 8h p.i., scale bar = 20 µm, and (B) Immunofluorescence at 16h p.i., scale bar = 50 µm. Single channel images were converted to grayscale. (C) Immunoblots showing changes in tight junction proteins during infection with *Nm* (MOI 10). Protein samples were collected from iPSC-BECs plated on standard tissue culture plates at 4, 8, 24 and 32h p.i. with or without *Nm* challenge. Arrows point to potential modifications of Occludin generated upon MC58 infection: grey arrow - potential cleavage product; black arrows - potential post-translational modification. Densitometry normalized to Actin is shown for comparison. Data shows mean ± S.D. of three independent experiments.

**Supplementary Figure 4. *Nm* infection leads to inflammatory activation of iPSC-BECs.** (A) Detection of RANTES, IFN-γ and IL-8 in supernatants of mock-, MC58Δ*siaD*- and 8013/12-infected iPSC-BEC monolayers by Luminex bead-based multiplex assays during a time course of 2, 4, 6, 8 and 24h of infection. (B) qPCR measurement of *CCL5* and *IFNG* transcript levels in iPSC-BECs with and without *Nm* challenge at 8 and 24h of infection. (C) Detection of VCAM-1, E-Selectin, MCP-1 and Gro-β in supernatants of mock-, MC58-, MC58Δ*siaD*- and 8013/12-infected iPSC-BEC monolayers as described for panel A. (D) Time-dependent secretion of IL-8 and IL-6 by HBMECs in response to *Nm* infection detected by Luminex bead-based multiplex assays. Data show mean ± S.E.M of three independent experiments done in duplicate. For A, B, and D Student’s *t* test was used to determine significance. For C Student’s *t* test was used to determine significant changes between 8013/12-infected cells and mock-treated controls and two-way ANOVA was used to determine significant changes between MC58-infected cells, MC58Δ*siaD*-infected cells and mock-treated controls. *, p < 0.05; **, p < 0.01; ****, p < 0.0001.

**Supplementary Figure 5. FACS analysis of infected iPSC-BECs.** (A) Representative FACS plots of a timecourse of infection of iPSC-BECs with GFP-expressing *Nm* (MOI 10). The cells were gated on the GFP (intracellular *Nm*) and PE (as a proxy of a cell’s auto-fluorescence) channels. The percentage values of iPSC-BECs containing intracellular *Nm* (i.e. GFP-positive events) are given. For RNA-Seq analysis, the GFP-positive fraction at the 24h time point was sort-collected and separated from non-infected bystander iPSC-BECs (i.e. GFP-negative events). The respective mock control (also sorted for the GFP-negative population) was included as a reference. (B) Heat map showing differentially expressed non-coding RNAs at 24h of infection. Plotted are all genes that were significantly differentially expressed (adjusted *P*-value < 0.1; DESeq2). Sequencing data is derived from two biological replicates.
